# A binuclear Cu^II^/Ca^II^ thio­cyanate complex with a Schiff base ligand derived from *o*-vanillin and ammonia

**DOI:** 10.1107/S205698902000211X

**Published:** 2020-02-21

**Authors:** Nataliya Plyuta, Olga Yu. Vassilyeva, Vladimir N. Kokozay, Iryna Omelchenko, Svitlana Petrusenko

**Affiliations:** a Department of Inorganic Chemistry, Taras Shevchenko National University of Kyiv, Volodymyrska str. 64/13, 01601 Kyiv, Ukraine; b Laboratoire MOLTECH-Anjou UMR 6200, UFR Sciences, CNRS, Université d’Angers, Bat. K, 2 Bd. Lavoisier, 49045 Angers, France; c Institute for Single Crystals, National Academy of Sciences of Ukraine, Nauky ave. 60, Kharkiv 61001, Ukraine

**Keywords:** crystal structure, Schiff base ligand, copper, calcium, heterometallic, hydrogen bond

## Abstract

The new heterometallic complex, aqua-1κ*O*-bis­(μ_2_-2-imino­methyl-6-meth­oxy­phenolato-1κ^2^ *O* ^1^,*O* ^6^:2κ^2^ *O* ^1^,*N*)bis­(thio­cyanato-1κ*N*)calcium(II)copper(II), [CaCu(C_8_H_8_NO_2_)_2_(NCS)_2_(H_2_O)], has been synthesized using a one-pot reaction of copper powder, calcium oxide, *o*-vanillin and ammonium thio­cyanate in methanol under ambient conditions. The Schiff base ligand (C_8_H_9_NO_2_) is generated *in situ* from the condensation of *o*-vanillin and ammonia, which is released from the initial NH_4_SCN. The title compound consists of a discrete binuclear mol­ecule with a {Cu(μ-O)_2_Ca} core, in which the Cu⋯Ca distance is 3.4275 (6) Å. The coordination geometries of the four-coordinate copper atom in the [CuN_2_O_2_] chromophore and the seven-coordinate calcium atom in the [CaO_5_N_2_] chromophore can be described as distorted square planar and penta­gonal bipyramidal, respectively. In the crystal, O—H⋯S hydrogen bonds between the coordinating water mol­ecules and thio­cyanate groups form a supra­molecular chain with a zigzag-shaped calcium skeleton.

## Abstract 2

A new title heterometallic Cu/Ca complex, [Cu(C_8_H_8_NO_2_)_2_Ca(NCS)_2_(H_2_O)], with a Schiff base ligand derived from *o*-vanillin and ammonia has been synthesized by a facile one-pot reaction under ambient conditions. In the crystal, O—H⋯S hydrogen bonds between the coordinating water mol­ecules and thio­cyanate groups form a supra­molecular chain with a zigzag-shaped calcium skeleton.

## Chemical context   

The coordination chemistry of *s*-block elements is a fairly new and rapidly growing area of research (Fromm, 2008 ▸). Among the many systems studied, special attention is paid to heterometallic Cu/Ca complexes because of their structural diversity, relatively low toxicity, useful properties such as catalytic (Saha *et al.*, 2016 ▸; Liu *et al.*, 2017 ▸; Mon *et al.*, 2016 ▸), magnetic (Sanchis *et al.*, 1992 ▸; Zhang *et al.*, 2013 ▸), luminescent (Zou & Gao, 2016 ▸), sorption (Grancha *et al.*, 2017 ▸) and bioactivity (Mon *et al.*, 2018 ▸; Grancha *et al.*, 2016 ▸), and therefore high potential for applications. In the course of our systematic work on the development of the ‘direct synthesis’ (DS) approach, we have been successful in preparing different homo- and heterometallic complexes with transition metals (Kokozay *et al.*, 2018 ▸). Herein we report the synthesis and crystal structure of the title compound.
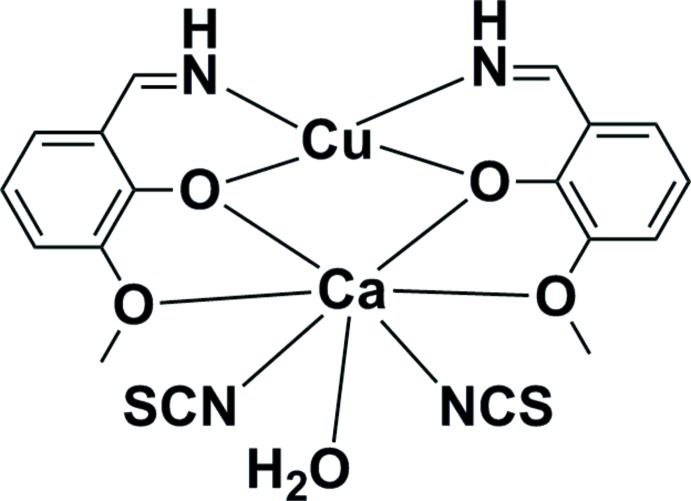


## Structural commentary   

**Figure 1:**
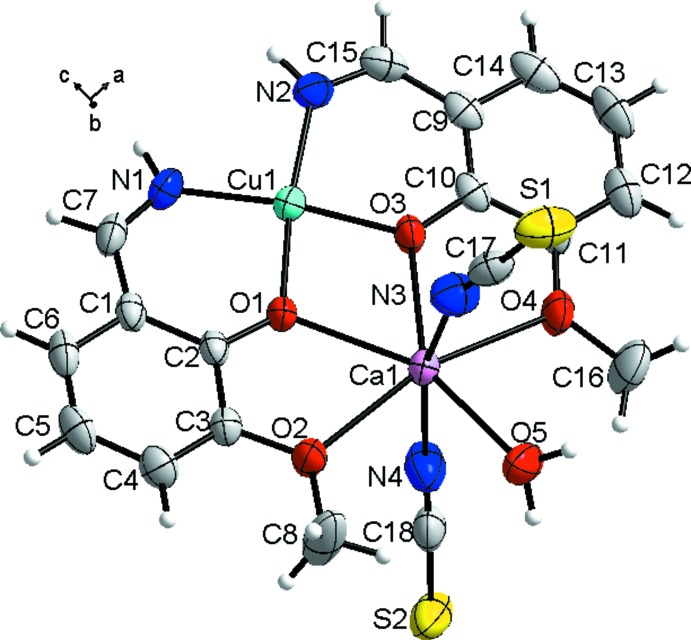
Mol­ecular structure of the title compound, with the numbering scheme and displacement ellipsoids drawn at the 50% probability level.

**Figure 2:**
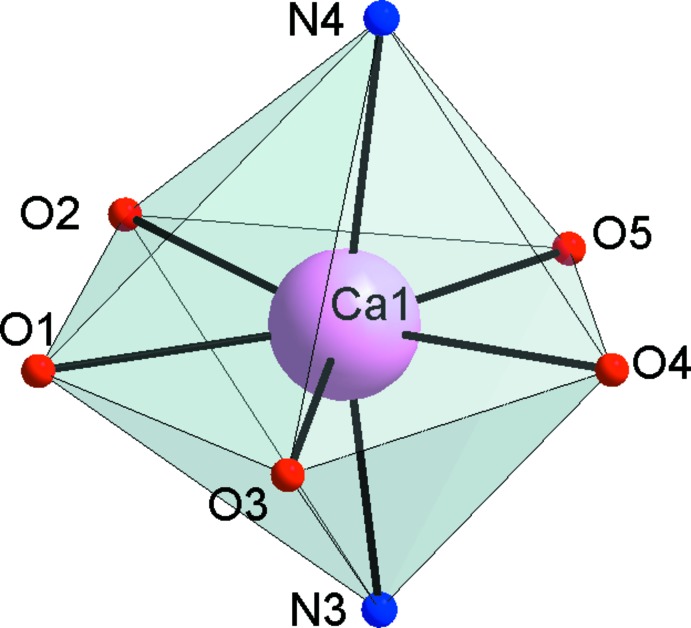
Coordination polyhedron of the calcium atom in the title compound.

The main structural unit is the heterometallic mol­ecular complex formed by divalent copper and calcium ions with two deprotonated Schiff base ligands (*L* ^−^ = C_8_H_8_NO_2_^−^), two thio­cyanate ions and one water mol­ecule (Fig. 1 ▸). The metal atoms are joined through two μ-O bridges from the phenolato-groups of the organic ligands, giving a binuclear {Cu(μ-O)_2_Ca} core with a Cu⋯Ca distance of 3.4275 (6) Å and Cu—O—Ca angles of 106.15 (8) and 106.64 (8)°. The copper atom is four-coordinated by two imino N and two phenoxo O atoms from the Schiff base ligands. The coordination geometry of the CuN_2_O_2_ chromophore is slightly distorted square planar; the Cu—O and Cu—N bond lengths vary in the range of 1.918 (2)–1.937 (2) Å and the corresponding *cis*/*trans* bond angles deviate from ideal symmetry by less than 8° with *τ*_4_ = 0.112 (Yang *et al.*, 2007 ▸). The copper atom is displaced from the N_2_O_2_ plane by *ca* 0.01 Å. All of the O atoms of the {Cu(*L*)_2_} moiety chelate the calcium atom in a tetra­dentate manner and the coordination sphere of the Ca center is further completed by two SCN groups and one water mol­ecule giving a coordination number of seven. The CaO_5_N_2_ chromophore can be described as having a distorted penta­gonal–bipyramidal geometry with the oxygen atoms in the equatorial plane and the nitro­gen atoms in the axial positions (Fig. 2 ▸). The calcium atom is located on the least-squares plane through the five equatorial O atoms, the sum of all O—Ca—O *cis* angles being 361°. The longest Ca—O bond distances [2.511 (2) and 2.521 (2) Å] are observed for the coordinating meth­oxy groups and the shortest ones [2.339 (2)–2.356 (2) Å] for the phenoxido groups and the water mol­ecule. The values are in accordance with those found in related binuclear Cu/Ca complexes (Mondal *et al.*, 2011 ▸; Constable *et al.*, 2010 ▸; Chandrasekhar *et al.*, 2012 ▸). The Cu⋯Ca separation [3.4275 (6) Å] is inter­mediate compared to the analogous distances of 3.363 and 3.462 Å, respectively, in [Cu*L*Ca(ClO_4_)_2_(H_2_O)] (Mondal *et al.*, 2011 ▸) and [*L*CuCa(NO_3_)_2_] (Chandrasekhar *et al.*, 2012 ▸). The *N*,*O*,*O*,*O′*-tetra­dentate coordination mode, or [2.11_2_1] in the Harris notation (Coxall *et al.*, 2000 ▸), of the H*L* ligand has been observed previously in [Ni(*L*)_2_Na(ClO_4_)(H_2_O)] (Costes *et al.*, 1994 ▸). The bond-valence-sum (BVS) analysis applied to the corresponding bond lengths leads to the +2 oxidation state for both metals: 2.07 (Cu) and 2.11 (Ca) (Brown & Altermatt, 1985 ▸; Chen & Adams, 2017 ▸).

## Supra­molecular features   

**Figure 3:**
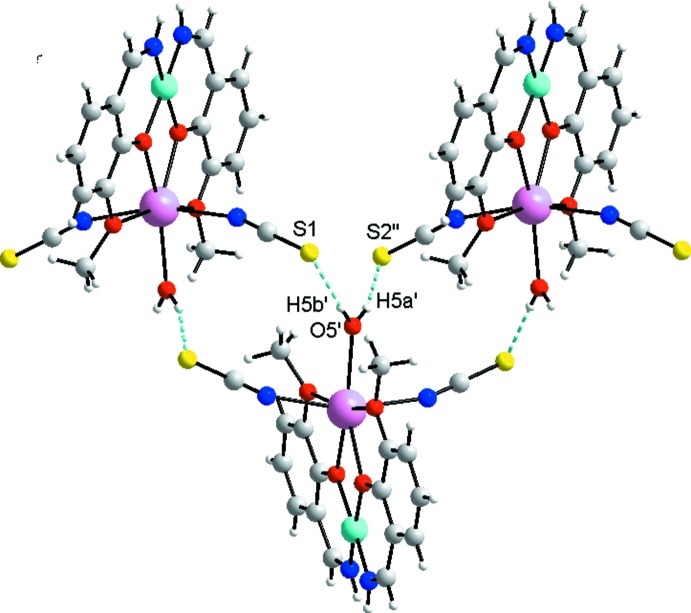
Packing diagram of the title compound, showing inter­molecular O—H⋯S hydrogen bonds forming a chain structure. [Symmetry codes: (i) *x* − 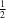, −*y* + 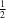, *z* + 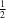; (ii) −*x*, 1 − *y*, −*z*.]

**Figure 4:**
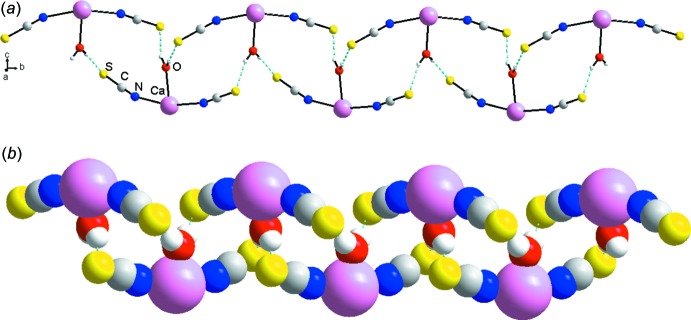
Fragment of the crystal structure of the title compound, illustrating the chain skeleton based on the [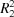
(12)]_2_ synthon in (*a*) ball-and-stick and (*b*) space-filling mode.

**Figure 5:**
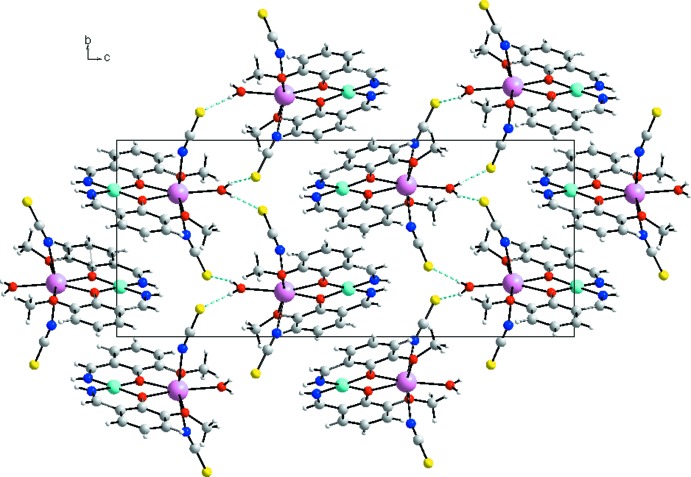
Packing diagram of the title compound viewed along the *a* axis, showing the supra­molecular chains. The dashed lines denote hydrogen bonds.

The coordinating water mol­ecule and thio­cyanate ions of each binuclear complex are involved in four O—H⋯S hydrogen bonds (Table 1 ▸) with two adjacent complexes. The hydrogen-bonded repeat unit can be described as a double twelve-membered ring motif [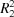
(12)]_2_ (Bernstein *et al.*, 1995 ▸) (Fig. 3 ▸). A fragment of the crystal structure showing the chain skeleton based on the [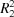
(12)]_2_ synthon is shown in Fig. 4 ▸. It should be noted that the arrangement of calcium atoms within the chain has a zigzag shape with all metal atoms lying in the same plane. The shortest Ca⋯Ca distance is 7.792 (7) Å and the angle formed by the three nearest metal centers is 85.093 (7)°. The supra­molecular chains run parallel to the *b*-axis (Fig. 5 ▸). Weak N—H⋯S hydrogen bonds (Table 1 ▸) and a π–π stacking inter­action between the C1–C6 ring and the adjacent C9–C14(*x* − 1, *y*, *z*) ring [dihedral angle between the rings 4.6 (1)°, mean inter­planar separation 3.40 Å and plane shift 0.69 (1) Å] link neighbouring chains, increasing the whole dimensionality of the crystal framework.

## Database survey   

To date, the crystal structures of 72 complexes containing copper and calcium are known (CSD, version 5.40, last update February 2019; Groom *et al.*, 2016 ▸). Most of them possess polymeric or ionic frameworks. Only five examples were found of mol­ecular binuclear Cu/Ca complexes, including two formed by carboxyl­ate ligands (Smith *et al.*, 1985 ▸; Breeze & Wang, 1994 ▸) and three with symmetric salen-type Schiff base ligands (Constable *et al.*, 2010 ▸; Mondal *et al.*, 2011 ▸; Chandrasekhar *et al.*, 2012 ▸). To the best of our knowledge, [Cu(*L*)_2_Ca(NCS)_2_(H_2_O)] is the first mol­ecular binuclear Cu/Ca complex with an asymmetric Schiff base ligand to have been characterized crystallographically.

## Synthesis and crystallization   

The following system has been investigated:

Cu^0^–CaO–*o*-vanillin–NH_4_SCN–methanol (open air),

and the heterometallic complex [Cu(*L*)_2_Ca(NCS)_2_(H_2_O)] was obtained. Its formation can be described by the following scheme:

Cu^0^ + CaO + 2*o*-vanillin + 2NH_4_SCN + 1/2O_2(air)_→ [Cu(L)_2_Ca(NCS)_2_(H_2_O)] + 3H_2_O,

where the Schiff base H*L* can be regarded as a product of the condensation of *o*-vanillin and NH_3_, which is released from NH_4_SCN in the basic environment.

Copper powder (0.06 g, 1 mmol), CaO (0.11 g, 2 mmol), *o*-vanillin (0.3 g, 2 mmol) and NH_4_SCN (0.15 g, 2 mmol) were added to 30 ml of methanol. The reaction mixture was stirred magnetically at 323–333 K for *ca* 5 h until the complete dissolution of the copper powder was observed. The solution was filtered and left for 1 d, and then light-orange crystals were formed. Yield: 0.26 g (48.3%, Cu). Analysis calculated for CaCuC_18_N_4_H_18_O_5_S_2_: Ca 7.45, Cu 11.81, C 40.18, N 10.41, H 3.37, S 11.92. Found: Ca 8.1, Cu 11.2, C 36.5, N 10.1, H 3.2, S 11.4. FT–IR (KBr, ν_max_ cm^−1^): 3349 *vs*, 3187 *vs*, 2942 *s*, 2076 *vs*, 1617*vs*, 1555 *m*, 1464 *vs*, 1386 *s*, 1318 *s*, 1245 *s*, 1225 *vs*, 1162 *m*, 1074 *s*, 1036 *m*, 948 *m*, 853 *m*, 823 *m*, 738 *s*, 652 *m*, 617 *m*, 571 *m*, 515 *m*, 469 *m*.

## Refinement   

Crystal data, data collection and structure refinement details are summarized in Table 2 ▸. H atoms of CH and CH_3_ groups were placed in idealized positions (C—H = 0.93–0.96 Å) and constrained to ride on their parent atoms, with *U*_iso_(H) = 1.2*U*_eq_(C) for CH and 1.5*U*_eq_(C) for CH_3_. All H atoms of the NH and OH groups were located in a difference-Fourier map and refined isotropically; the N—H and O—H distances were restrained to have fixed lengths of 0.82 (1) and 0.85 (1) Å, respectively.

## Supplementary Material

Crystal structure: contains datablock(s) I. DOI: 10.1107/S205698902000211X/is5531sup1.cif

Structure factors: contains datablock(s) I. DOI: 10.1107/S205698902000211X/is5531Isup2.hkl

CCDC reference: 1984001

Additional supporting information:  crystallographic information; 3D view; checkCIF report

## Figures and Tables

**Table 1:** Hydrogen-bond geometry (Å, °)

*D*—H⋯*A*	*D*—H	H⋯*A*	*D*⋯*A*	*D*—H⋯*A*
O5—H5*A*⋯S2^i^	0.85 (1)	2.47 (1)	3.297 (3)	169 (4)
O5—H5*B*⋯S1^ii^	0.84 (1)	2.40 (2)	3.226 (3)	166 (5)
N1—H1⋯S2^iii^	0.82 (1)	2.80 (2)	3.500 (2)	145 (3)
N2—H2⋯S1^iv^	0.82 (1)	2.61 (1)	3.403 (3)	163 (3)

Symmetry codes: (i) 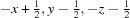; (ii) 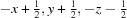; (iii) 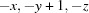; (iv) 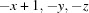.

**Table 2:** Experimental details

Crystal data	
Chemical formula	[CaCu(C_8_H_8_NO_2_)_2_(NCS)_2_(H_2_O)]
*M*_r_	538.10
Crystal system, space group	Monoclinic, *P*2_1_/*n*
Temperature (K)	298
*a*, *b*, *c* (Å)	8.5623 (3), 10.5377 (3), 24.4439 (7)
β (°)	90.768 (3)
*V* (Å^3^)	2205.30 (13)
*Z*	4
Radiation type	Mo *K*α
μ (mm^−1^)	1.45
Crystal size (mm)	0.40 × 0.20 × 0.04

Data collection	
Diffractometer	Oxford Diffraction Xcalibur, Sapphire3
Absorption correction	Multi-scan (*CrysAlis PRO*; Oxford Diffraction, 2007 ▸)
*T*_min_, *T*_max_	0.671, 1.000
No. of measured, independent and observed [*I* > 2σ(*I*)] reflections	15599, 5844, 3849
*R*_int_	0.037
(sin θ/λ)_max_ (Å^−1^)	0.712

Refinement	
*R*[*F* ^2^ > 2σ(*F* ^2^)], *wR*(*F* ^2^), *S*	0.044, 0.103, 1.02
No. of reflections	5844
No. of parameters	298
No. of restraints	4
H-atom treatment	H atoms treated by a mixture of independent and constrained refinement
Δρ_max_, Δρ_min_ (e Å^−3^)	0.55, −0.53

Computer programs: *CrysAlis PRO* (Oxford Diffraction, 2007 ▸), SHELXT (Sheldrick, 2015*a* ▸), *SHELXL2016/6* (Sheldrick, 2015*b* ▸) and *OLEX2* (Dolomanov *et al.*, 2009 ▸).

## supplementary crystallographic information

### Crystal data

**Table:**

[CaCu(C_8_H_8_NO_2_)_2_(NCS)_2_(H_2_O)]	*F*(000) = 1100
*M**_r_* = 538.10	*D*_x_ = 1.621 Mg m^−^^3^
Monoclinic, *P*2_1_/*n*	Mo *K*α radiation, λ = 0.71073 Å
*a* = 8.5623 (3) Å	Cell parameters from 3455 reflections
*b* = 10.5377 (3) Å	θ = 3.2–28.2°
*c* = 24.4439 (7) Å	µ = 1.45 mm^−^^1^
β = 90.768 (3)°	*T* = 298 K
*V* = 2205.30 (13) Å^3^	Plate, clear light orange
*Z* = 4	0.40 × 0.20 × 0.04 mm

### Data collection

**Table:**

Oxford Diffraction Xcalibur, Sapphire3 diffractometer	5844 independent reflections
Radiation source: Enhance (Mo) X-ray Source	3849 reflections with *I* > 2σ(*I*)
Graphite monochromator	*R*_int_ = 0.037
Detector resolution: 16.1827 pixels mm^-1^	θ_max_ = 30.4°, θ_min_ = 3.1°
ω scans	*h* = −11→11
Absorption correction: multi-scan (CrysAlisPro; Oxford Diffraction, 2007)	*k* = −14→13
*T*_min_ = 0.671, *T*_max_ = 1.000	*l* = −31→33
15599 measured reflections	

### Refinement

**Table:**

Refinement on *F*^2^	Primary atom site location: structure-invariant direct methods
Least-squares matrix: full	Hydrogen site location: mixed
*R*[*F*^2^ > 2σ(*F*^2^)] = 0.044	H atoms treated by a mixture of independent and constrained refinement
*wR*(*F*^2^) = 0.103	*w* = 1/[σ^2^(*F*_o_^2^) + (0.0412*P*)^2^ + 0.4639*P*] where *P* = (*F*_o_^2^ + 2*F*_c_^2^)/3
*S* = 1.02	(Δ/σ)_max_ < 0.001
5844 reflections	Δρ_max_ = 0.55 e Å^−^^3^
298 parameters	Δρ_min_ = −0.53 e Å^−^^3^
4 restraints	

### Special details

**Table:**

Geometry. All esds (except the esd in the dihedral angle between two l.s. planes) are estimated using the full covariance matrix. The cell esds are taken into account individually in the estimation of esds in distances, angles and torsion angles; correlations between esds in cell parameters are only used when they are defined by crystal symmetry. An approximate (isotropic) treatment of cell esds is used for estimating esds involving l.s. planes.

### Fractional atomic coordinates and isotropic or equivalent isotropic displacement parameters (Å^2^)

**Table:**

	*x*	*y*	*z*	*U*_iso_*/*U*_eq_	
Cu1	0.25895 (4)	0.25464 (3)	0.00673 (2)	0.03322 (10)	
Ca1	0.22640 (7)	0.27119 (5)	−0.13299 (2)	0.03249 (14)	
S1	0.51771 (13)	−0.14153 (9)	−0.18147 (3)	0.0646 (3)	
S2	−0.03690 (13)	0.68682 (9)	−0.19653 (3)	0.0614 (3)	
O1	0.1048 (2)	0.22468 (17)	−0.04945 (7)	0.0338 (4)	
O2	−0.0464 (2)	0.1848 (2)	−0.13976 (7)	0.0414 (5)	
O3	0.3808 (2)	0.30690 (18)	−0.05483 (7)	0.0355 (4)	
O4	0.4805 (2)	0.3859 (2)	−0.14838 (7)	0.0450 (5)	
O5	0.2298 (3)	0.2535 (3)	−0.22876 (9)	0.0619 (7)	
H5A	0.305 (3)	0.225 (4)	−0.2470 (16)	0.098 (16)*	
H5B	0.179 (5)	0.288 (4)	−0.2545 (14)	0.119 (18)*	
N1	0.1121 (3)	0.2106 (2)	0.06324 (9)	0.0428 (6)	
H1	0.134 (4)	0.218 (3)	0.0957 (5)	0.055 (10)*	
N2	0.4350 (3)	0.2754 (2)	0.05565 (10)	0.0409 (6)	
H2	0.430 (4)	0.254 (3)	0.0876 (5)	0.044 (9)*	
N3	0.3366 (4)	0.0593 (3)	−0.14345 (10)	0.0554 (7)	
N4	0.1238 (3)	0.4817 (3)	−0.15087 (11)	0.0631 (8)	
C1	−0.0978 (3)	0.1323 (2)	0.00536 (10)	0.0331 (6)	
C2	−0.0321 (3)	0.1693 (2)	−0.04459 (10)	0.0291 (6)	
C3	−0.1196 (3)	0.1456 (2)	−0.09263 (10)	0.0323 (6)	
C4	−0.2633 (4)	0.0899 (3)	−0.09111 (12)	0.0424 (7)	
H4	−0.319638	0.076917	−0.123429	0.051*	
C5	−0.3257 (4)	0.0525 (3)	−0.04175 (13)	0.0454 (7)	
H5	−0.422967	0.013284	−0.040983	0.054*	
C6	−0.2446 (3)	0.0731 (3)	0.00573 (12)	0.0417 (7)	
H6	−0.286972	0.047557	0.038788	0.050*	
C7	−0.0217 (4)	0.1594 (3)	0.05695 (11)	0.0401 (7)	
H7	−0.074932	0.137499	0.088485	0.048*	
C8	−0.1315 (5)	0.1643 (5)	−0.18992 (12)	0.0846 (15)	
H8A	−0.073501	0.198512	−0.219853	0.127*	
H8B	−0.231122	0.205765	−0.188105	0.127*	
H8C	−0.146754	0.074931	−0.195367	0.127*	
C9	0.6152 (3)	0.3727 (3)	−0.00718 (11)	0.0374 (6)	
C10	0.5218 (3)	0.3585 (2)	−0.05441 (11)	0.0335 (6)	
C11	0.5812 (3)	0.4028 (3)	−0.10421 (11)	0.0377 (6)	
C12	0.7263 (4)	0.4563 (3)	−0.10718 (13)	0.0470 (7)	
H12	0.763901	0.483621	−0.140689	0.056*	
C13	0.8170 (4)	0.4696 (3)	−0.06040 (15)	0.0530 (8)	
H13	0.915615	0.506000	−0.062517	0.064*	
C14	0.7631 (4)	0.4299 (3)	−0.01150 (14)	0.0486 (8)	
H14	0.824788	0.440632	0.019761	0.058*	
C15	0.5658 (4)	0.3266 (3)	0.04523 (12)	0.0428 (7)	
H15	0.636341	0.335062	0.074278	0.051*	
C16	0.5129 (5)	0.4603 (4)	−0.19647 (12)	0.0657 (11)	
H16A	0.427580	0.452705	−0.222133	0.099*	
H16B	0.606992	0.430087	−0.212968	0.099*	
H16C	0.525799	0.547751	−0.186305	0.099*	
C17	0.4078 (4)	−0.0245 (3)	−0.15950 (11)	0.0442 (7)	
C18	0.0590 (4)	0.5668 (3)	−0.17037 (11)	0.0461 (8)	

### Atomic displacement parameters (Å^2^)

**Table:**

	*U*^11^	*U*^22^	*U*^33^	*U*^12^	*U*^13^	*U*^23^
Cu1	0.03093 (18)	0.0417 (2)	0.02712 (16)	0.00156 (16)	0.00306 (13)	0.00148 (13)
Ca1	0.0298 (3)	0.0411 (3)	0.0267 (3)	−0.0003 (3)	0.0050 (2)	0.0035 (2)
S1	0.0866 (8)	0.0591 (5)	0.0475 (5)	0.0227 (5)	−0.0176 (5)	−0.0163 (4)
S2	0.0702 (6)	0.0635 (6)	0.0508 (5)	0.0184 (5)	0.0145 (4)	0.0176 (4)
O1	0.0275 (10)	0.0456 (11)	0.0284 (9)	−0.0035 (9)	0.0037 (7)	0.0061 (8)
O2	0.0356 (11)	0.0576 (12)	0.0310 (9)	−0.0097 (11)	0.0004 (8)	0.0014 (9)
O3	0.0286 (10)	0.0461 (11)	0.0319 (9)	−0.0072 (9)	0.0057 (8)	−0.0013 (8)
O4	0.0430 (13)	0.0508 (12)	0.0416 (11)	−0.0128 (11)	0.0101 (9)	0.0079 (9)
O5	0.0562 (16)	0.098 (2)	0.0315 (11)	0.0175 (16)	0.0059 (11)	0.0061 (12)
N1	0.0490 (17)	0.0540 (15)	0.0256 (12)	0.0007 (14)	0.0063 (11)	0.0032 (11)
N2	0.0413 (15)	0.0484 (15)	0.0328 (13)	0.0064 (13)	−0.0028 (11)	−0.0018 (11)
N3	0.0602 (19)	0.0542 (17)	0.0516 (16)	0.0112 (16)	−0.0036 (14)	−0.0043 (13)
N4	0.0536 (19)	0.0588 (18)	0.0773 (19)	0.0139 (16)	0.0206 (16)	0.0210 (15)
C1	0.0294 (15)	0.0306 (13)	0.0396 (14)	0.0071 (12)	0.0115 (12)	0.0051 (11)
C2	0.0244 (13)	0.0282 (13)	0.0349 (13)	0.0041 (12)	0.0075 (11)	0.0037 (10)
C3	0.0309 (15)	0.0306 (13)	0.0355 (14)	0.0002 (12)	0.0072 (11)	0.0004 (11)
C4	0.0379 (17)	0.0399 (16)	0.0494 (17)	−0.0050 (15)	0.0021 (14)	−0.0033 (13)
C5	0.0311 (16)	0.0379 (16)	0.067 (2)	−0.0076 (14)	0.0095 (15)	−0.0018 (14)
C6	0.0367 (17)	0.0340 (15)	0.0549 (17)	0.0011 (14)	0.0206 (14)	0.0076 (13)
C7	0.0454 (18)	0.0405 (16)	0.0348 (14)	0.0052 (15)	0.0165 (13)	0.0083 (12)
C8	0.077 (3)	0.145 (4)	0.0323 (17)	−0.051 (3)	−0.0041 (17)	0.000 (2)
C9	0.0307 (15)	0.0302 (14)	0.0514 (17)	0.0064 (13)	0.0017 (13)	−0.0072 (12)
C10	0.0275 (14)	0.0276 (13)	0.0455 (15)	0.0045 (12)	0.0047 (12)	−0.0062 (11)
C11	0.0341 (16)	0.0314 (14)	0.0480 (16)	0.0001 (13)	0.0096 (13)	−0.0019 (12)
C12	0.0376 (18)	0.0366 (16)	0.067 (2)	−0.0022 (15)	0.0147 (15)	0.0010 (14)
C13	0.0307 (17)	0.0382 (17)	0.090 (2)	−0.0075 (15)	0.0083 (17)	−0.0027 (17)
C14	0.0344 (17)	0.0359 (16)	0.075 (2)	0.0037 (15)	−0.0089 (16)	−0.0128 (15)
C15	0.0403 (18)	0.0444 (17)	0.0432 (16)	0.0107 (15)	−0.0114 (14)	−0.0115 (13)
C16	0.080 (3)	0.068 (2)	0.0498 (19)	−0.018 (2)	0.0130 (18)	0.0179 (17)
C17	0.053 (2)	0.0468 (18)	0.0321 (14)	−0.0011 (17)	−0.0102 (14)	−0.0010 (13)
C18	0.0421 (18)	0.0538 (19)	0.0426 (16)	−0.0008 (16)	0.0158 (14)	0.0026 (14)

### Geometric parameters (Å, º)

**Table:**

Cu1—Ca1	3.4275 (6)	C1—C2	1.406 (3)
Cu1—O1	1.9183 (18)	C1—C6	1.404 (4)
Cu1—O3	1.9232 (18)	C1—C7	1.440 (4)
Cu1—N1	1.937 (2)	C2—C3	1.407 (3)
Cu1—N2	1.924 (2)	C3—C4	1.364 (4)
Ca1—O1	2.3565 (17)	C4—H4	0.9300
Ca1—O2	2.511 (2)	C4—C5	1.383 (4)
Ca1—O3	2.3394 (18)	C5—H5	0.9300
Ca1—O4	2.521 (2)	C5—C6	1.362 (4)
Ca1—O5	2.349 (2)	C6—H6	0.9300
Ca1—N3	2.439 (3)	C7—H7	0.9300
Ca1—N4	2.423 (3)	C8—H8A	0.9600
S1—C17	1.646 (3)	C8—H8B	0.9600
S2—C18	1.633 (4)	C8—H8C	0.9600
O1—C2	1.315 (3)	C9—C10	1.403 (4)
O2—C3	1.382 (3)	C9—C14	1.408 (4)
O2—C8	1.434 (3)	C9—C15	1.439 (4)
O3—C10	1.324 (3)	C10—C11	1.405 (4)
O4—C11	1.384 (3)	C11—C12	1.367 (4)
O4—C16	1.443 (3)	C12—H12	0.9300
O5—H5A	0.845 (10)	C12—C13	1.381 (4)
O5—H5B	0.844 (10)	C13—H13	0.9300
N1—H1	0.815 (10)	C13—C14	1.353 (4)
N1—C7	1.274 (4)	C14—H14	0.9300
N2—H2	0.816 (10)	C15—H15	0.9300
N2—C15	1.272 (4)	C16—H16A	0.9600
N3—C17	1.145 (4)	C16—H16B	0.9600
N4—C18	1.155 (4)	C16—H16C	0.9600
			
O1—Cu1—Ca1	41.33 (5)	C17—N3—Ca1	161.8 (3)
O1—Cu1—O3	82.10 (8)	C18—N4—Ca1	163.1 (3)
O1—Cu1—N1	91.37 (10)	C2—C1—C7	121.6 (3)
O1—Cu1—N2	171.72 (9)	C6—C1—C2	119.8 (2)
O3—Cu1—Ca1	40.84 (5)	C6—C1—C7	118.5 (2)
O3—Cu1—N1	172.27 (10)	O1—C2—C1	124.7 (2)
O3—Cu1—N2	91.42 (10)	O1—C2—C3	118.0 (2)
N1—Cu1—Ca1	132.69 (8)	C1—C2—C3	117.4 (2)
N2—Cu1—Ca1	131.76 (8)	O2—C3—C2	113.6 (2)
N2—Cu1—N1	95.45 (11)	C4—C3—O2	124.8 (2)
O1—Ca1—Cu1	32.52 (4)	C4—C3—C2	121.6 (2)
O1—Ca1—O2	63.89 (6)	C3—C4—H4	119.8
O1—Ca1—O4	128.41 (6)	C3—C4—C5	120.3 (3)
O1—Ca1—N3	94.38 (8)	C5—C4—H4	119.8
O1—Ca1—N4	100.56 (8)	C4—C5—H5	120.0
O2—Ca1—Cu1	96.33 (4)	C6—C5—C4	120.0 (3)
O2—Ca1—O4	165.45 (6)	C6—C5—H5	120.0
O3—Ca1—Cu1	32.52 (4)	C1—C6—H6	119.6
O3—Ca1—O1	64.99 (6)	C5—C6—C1	120.8 (3)
O3—Ca1—O2	128.85 (6)	C5—C6—H6	119.6
O3—Ca1—O4	64.24 (6)	N1—C7—C1	125.8 (3)
O3—Ca1—O5	144.32 (8)	N1—C7—H7	117.1
O3—Ca1—N3	91.01 (8)	C1—C7—H7	117.1
O3—Ca1—N4	101.51 (9)	O2—C8—H8A	109.5
O4—Ca1—Cu1	96.56 (4)	O2—C8—H8B	109.5
O5—Ca1—Cu1	170.80 (8)	O2—C8—H8C	109.5
O5—Ca1—O1	149.18 (9)	H8A—C8—H8B	109.5
O5—Ca1—O2	85.97 (8)	H8A—C8—H8C	109.5
O5—Ca1—O4	82.36 (8)	H8B—C8—H8C	109.5
O5—Ca1—N3	79.22 (9)	C10—C9—C14	119.1 (3)
O5—Ca1—N4	84.39 (10)	C10—C9—C15	121.7 (3)
N3—Ca1—Cu1	91.80 (6)	C14—C9—C15	119.1 (3)
N3—Ca1—O2	91.27 (9)	O3—C10—C9	124.0 (2)
N3—Ca1—O4	95.02 (9)	O3—C10—C11	118.0 (2)
N4—Ca1—Cu1	104.52 (7)	C9—C10—C11	117.9 (3)
N4—Ca1—O2	89.15 (9)	O4—C11—C10	113.8 (2)
N4—Ca1—O4	81.12 (9)	C12—C11—O4	124.7 (3)
N4—Ca1—N3	163.53 (9)	C12—C11—C10	121.5 (3)
Cu1—O1—Ca1	106.15 (8)	C11—C12—H12	120.0
C2—O1—Cu1	127.85 (15)	C11—C12—C13	120.0 (3)
C2—O1—Ca1	125.10 (15)	C13—C12—H12	120.0
C3—O2—Ca1	119.05 (15)	C12—C13—H13	119.8
C3—O2—C8	115.9 (2)	C14—C13—C12	120.4 (3)
C8—O2—Ca1	124.91 (18)	C14—C13—H13	119.8
Cu1—O3—Ca1	106.64 (8)	C9—C14—H14	119.5
C10—O3—Cu1	128.01 (16)	C13—C14—C9	121.1 (3)
C10—O3—Ca1	125.29 (16)	C13—C14—H14	119.5
C11—O4—Ca1	118.37 (15)	N2—C15—C9	126.2 (3)
C11—O4—C16	116.1 (2)	N2—C15—H15	116.9
C16—O4—Ca1	123.92 (19)	C9—C15—H15	116.9
Ca1—O5—H5A	125 (3)	O4—C16—H16A	109.5
Ca1—O5—H5B	134 (4)	O4—C16—H16B	109.5
H5A—O5—H5B	99 (4)	O4—C16—H16C	109.5
Cu1—N1—H1	122 (2)	H16A—C16—H16B	109.5
C7—N1—Cu1	127.4 (2)	H16A—C16—H16C	109.5
C7—N1—H1	111 (2)	H16B—C16—H16C	109.5
Cu1—N2—H2	121 (2)	N3—C17—S1	177.3 (3)
C15—N2—Cu1	127.5 (2)	N4—C18—S2	178.2 (3)
C15—N2—H2	111 (2)		
			
Cu1—O1—C2—C1	6.8 (4)	C3—C4—C5—C6	1.0 (4)
Cu1—O1—C2—C3	−173.44 (17)	C4—C5—C6—C1	0.1 (4)
Cu1—O3—C10—C9	6.7 (4)	C6—C1—C2—O1	−179.6 (2)
Cu1—O3—C10—C11	−172.68 (18)	C6—C1—C2—C3	0.6 (4)
Cu1—N1—C7—C1	−5.3 (4)	C6—C1—C7—N1	178.8 (3)
Cu1—N2—C15—C9	−4.8 (5)	C7—C1—C2—O1	3.6 (4)
Ca1—O1—C2—C1	174.37 (18)	C7—C1—C2—C3	−176.2 (2)
Ca1—O1—C2—C3	−5.8 (3)	C7—C1—C6—C5	175.9 (3)
Ca1—O2—C3—C2	4.8 (3)	C8—O2—C3—C2	−179.3 (3)
Ca1—O2—C3—C4	−175.7 (2)	C8—O2—C3—C4	0.2 (4)
Ca1—O3—C10—C9	−176.52 (19)	C9—C10—C11—O4	−179.4 (2)
Ca1—O3—C10—C11	4.1 (3)	C9—C10—C11—C12	1.2 (4)
Ca1—O4—C11—C10	−3.5 (3)	C10—C9—C14—C13	−0.9 (4)
Ca1—O4—C11—C12	175.9 (2)	C10—C9—C15—N2	−3.6 (5)
O1—C2—C3—O2	0.2 (3)	C10—C11—C12—C13	−1.1 (4)
O1—C2—C3—C4	−179.3 (2)	C11—C12—C13—C14	0.0 (5)
O2—C3—C4—C5	179.2 (3)	C12—C13—C14—C9	1.0 (5)
O3—C10—C11—O4	0.0 (4)	C14—C9—C10—O3	−179.5 (2)
O3—C10—C11—C12	−179.4 (2)	C14—C9—C10—C11	−0.1 (4)
O4—C11—C12—C13	179.5 (3)	C14—C9—C15—N2	178.5 (3)
C1—C2—C3—O2	−180.0 (2)	C15—C9—C10—O3	2.5 (4)
C1—C2—C3—C4	0.5 (4)	C15—C9—C10—C11	−178.1 (2)
C2—C1—C6—C5	−1.0 (4)	C15—C9—C14—C13	177.1 (3)
C2—C1—C7—N1	−4.3 (4)	C16—O4—C11—C10	162.7 (3)
C2—C3—C4—C5	−1.4 (4)	C16—O4—C11—C12	−17.9 (4)

### Hydrogen-bond geometry (Å, º)

**Table:**

*D*—H···*A*	*D*—H	H···*A*	*D*···*A*	*D*—H···*A*
O5—H5*A*···S2^i^	0.85 (1)	2.47 (1)	3.297 (3)	169 (4)
O5—H5*B*···S1^ii^	0.84 (1)	2.40 (2)	3.226 (3)	166 (5)
N1—H1···S2^iii^	0.82 (1)	2.80 (2)	3.500 (2)	145 (3)
N2—H2···S1^iv^	0.82 (1)	2.61 (1)	3.403 (3)	163 (3)

Symmetry codes: (i) −*x*+1/2, *y*−1/2, −*z*−1/2; (ii) −*x*+1/2, *y*+1/2, −*z*−1/2; (iii) −*x*, −*y*+1, −*z*; (iv) −*x*+1, −*y*, −*z*.
